# Porto-Sinusoidal Vascular Disease as the Cause of Portal Hypertension in Felty's Syndrome: A Case Report and Literature Review

**DOI:** 10.1155/2020/2618260

**Published:** 2020-07-01

**Authors:** Song Yang, Min Quan, Yue Li, Calvin Qian Pan, Huichun Xing

**Affiliations:** ^1^Center of Liver Diseases, Beijing Ditan Hospital of Capital Medical University, Beijing, China; ^2^Division of Gastroenterology and Hepatology, Department of Medicine, NYU Langone Health, New York University School of Medicine, New York 11355, USA

## Abstract

Felty's syndrome (FS) is a disorder wherein patients with rheumatoid arthritis develop splenomegaly, neutropenia, and in some cases, portal hypertension without underlying cirrhosis. Esophageal variceal bleeding is a complication of FS in patients with portal hypertension. In contrast to splenectomy, few reports exist on the management of variceal bleeding with endoscopic therapy. Moreover, the long-term outcome has not been reported. We present a patient with esophageal variceal bleeding due to portal hypertension secondary to Felty's syndrome. The patient was followed up for two years postendoscopy intervention. Literature review was performed and the histological features of portal hypertension in FS are discussed. The patient presented with a typical triad of rheumatoid arthritis (RA), splenomegaly, and neutropenia and was diagnosed as Felty's syndrome in 2012. She was admitted to our hospital in September 2017 for esophageal variceal bleeding. At the time of admission, her liver function test was normal. Abdominal CT showed no signs of cirrhosis and portal vein obstruction. Liver biopsy further excluded diagnosis of cirrhosis and supported the diagnosis of porto-sinusoidal vascular disease (PSVD), which was previously named as noncirrhotic idiopathic portal hypertension (NCIPH). An upper abdominal endoscopy revealed gastric and esophageal varices. A series of endoscopies was performed to ligate the esophageal varices. The patient was followed up for two years and did not show rebleeding. In conclusion, comorbid PSVD might be a cause of portal hypertension in FS patients. The present case had excellent outcome in two years, which supported the use of endoscopic therapy for the management of variceal bleeding in FS patients. Further large prospective study is needed to confirm the findings.

## 1. Introduction

Felty's syndrome (FS) is a rare clinical syndrome characterized by a triad of seropositive rheumatoid arthritis (RA), with severe joint involvement, splenomegaly, and neutropenia, which occurs in about 1% of RA patients. It was first described in 1924 by the American physician Augustus Roi Felty [1]. Diagnosis of FS is made when a patient meets these criteria: (1) classical or definite rheumatoid arthritis (ARA criteria), (2) splenomegaly detected by physical examination or radioisotope scan, (3) leucopenia (<4.0 × 10^9^/L) or neutropenia (<2.0 × 10^9^/L) or thrombocytopenia (<100 × l0^9^/L), and (4) no other known causes for cytopenia (e.g., drugs) or splenomegaly (e.g., lymphoma) [2]. No randomized clinical trials are available for FS, and no definitive recommendation can be made for the treatment for FS. Usually, methotrexate, corticosteroids, and hydroxychloroquine are used when the patient is first diagnosed. Case reports on rituximab and anti-TNF*α* agents showed promising efficacy. However, increased risk of infection and unsatisfactory long-term effects raise concerns for biological agents [3].

About 20% of FS patients showed portal hypertension and/or bleeding esophageal varices [4]. Pathogenesis of portal hypertension remains controversial. It is suggested that hepatic lesion, especially nodular regenerative hyperplasia may contribute to the portal hypertension [5]. Increased splenic blood flow may also lead to portal hypertension. There are several case reports suggesting that splenectomy might help to control the portal hypertension [6, 7]. However, there is no standard of care for esophageal varices in FS. Though there are reports that endoscopy could prevent fatal complications in patients with FS, long-term follow-up of patients who underwent endoscopic therapy is seldom reported [6]. Herein, we presented a case of FS with esophageal variceal bleeding. Liver biopsy indicated that porto-sinusoidal vascular disease (PSVD), which was previously named as noncirrhotic idiopathic portal hypertension (NCIPH) may contribute to the portal hypertension in FS. Also, the patient underwent endoscopic therapy for esophageal varices. Two-year follow-up showed no rebleeding. This case provided insights into the pathogenesis of portal hypertension in FS and the management of gastroesophageal varices in patients with FS.

## 2. Materials and Methods

### 2.1. Patient

A 48-year-old Chinese female presented to the emergency department with hematemesis and black stool (about 1000 mL), without abdominal pain on September 15, 2017. The patient showed mild palpitation and no syncope. Review of her past medical history revealed that in May 2012, the patient showed typical triad of rheumatoid arthritis (RA), splenomegaly, and neutropenia. The patient had normal liver function. Other causes of splenomegaly and neutropenia were excluded. The patient was first diagnosed as FS at the Peking Union Medical College Hospital. The patient started oral prednisone 40 mg qd in June 2012 as well as hydroxychloroquine 200 mg qd intermittently. Although symptoms of RA were alleviated, the splenomegaly and neutropenia persisted. In March 2017, the patient was switched from prednisone to methotrexate (detailed dosage unavailable) for uncontrolled neutropenia. The patient denied any history of other diseases or surgery. Also, the patient denied any history of alcohol and drug use. Moreover, no positive history of family members was reported. The physical examination revealed splenomegaly, anemic appearance, and multiple metacarpophalangeal joints and interphalangeal joints deformities.

### 2.2. Diagnostic Assessment

Liver function tests, complete blood count, viral hepatitis markers, autoimmune antibodies, and rheumatoid factor were tested when enrolled and during interval follow-up. Enhanced abdominal CT and portal vein reconstruction was performed to further clarify the causes of portal hypertension. Also, we did liver biopsy to exclude potential liver diseases.

### 2.3. Therapeutic Intervention

After hospital admission, the patient was given conventional treatment with intensive care, antibiotics, and hemostatic drugs. Endoscopy was performed and endoscopic injection sclerotherapy with polidocanol was performed to eliminate the varicosed vein. Carvedilol was not used in this patient for intolerance. The patient did not take splenectomy. The patient was followed-up every 6 months.

## 3. Results

**Figure 1:**
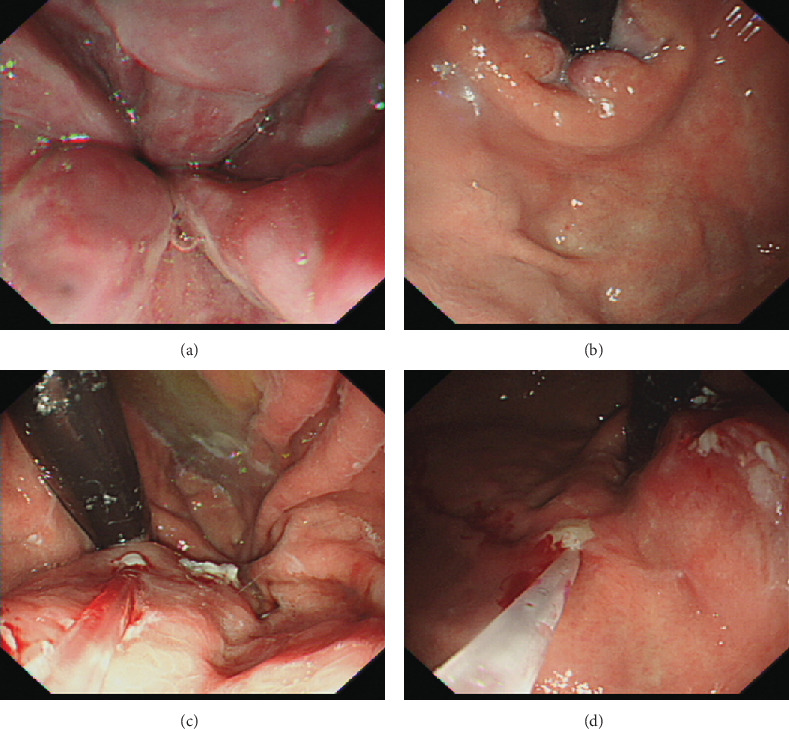
Endoscopy revealed type 1 gastroesophageal varices and F2 esophageal varices.

**Figure 2:**
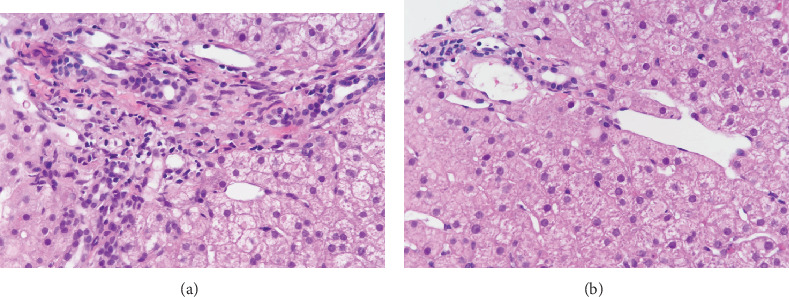
Liver biopsy revealed mild chronic hepatitis, no interface inflammation, hyperplasia of fibrous tissue with incomplete fiber interval formation, roughly normal bile duct, disappearance of portal vein in portal area (a) and enlarged and herniated portal vein (b).

**Figure 3:**
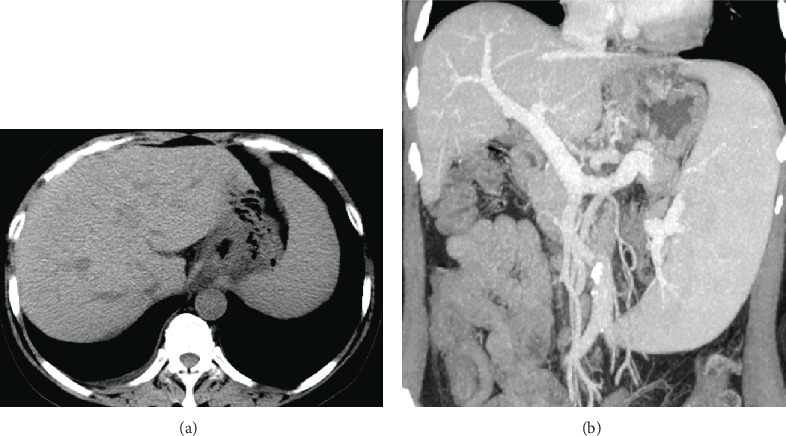
The abdominal enhanced CT showed no signs of cirrhosis. The splenic vein was significantly widened, and the spleen was enlarged.

Liver function tests, complete blood count, and rheumatoid factor are shown in Table 1. The patient showed no signs of hepatitis B and C, ANA was 1 : 1000 positive, ASMA (-), and AMA (-). Endoscopy when enrolled showed type 1 gastroesophageal varices (GOV1s) and F2 esophageal varices (Figure 1) [8]. Liver biopsy showed mild inflammation, no signs of cirrhosis. However, we found disappearance of portal vein in portal area (Figure 2(a)) and enlarged and herniated portal vein (Figure 2(b)) in biopsy, which are characterized signs of PSVD. Abdominal ultrasound showed normal liver morphology and splenomegaly (19 × 5.6 cm). Portal vein diameter was 14 mm with a blood velocity of 22 cm/s. Enhanced abdominal CT and portal vein reconstruction further confirmed splenomegaly and no signs of cirrhosis. No vascular embolization was found in portal vein reconstruction (Figure 3). The FibroScan liver stiffness is 12.6 Kpa.

**Figure 4:**
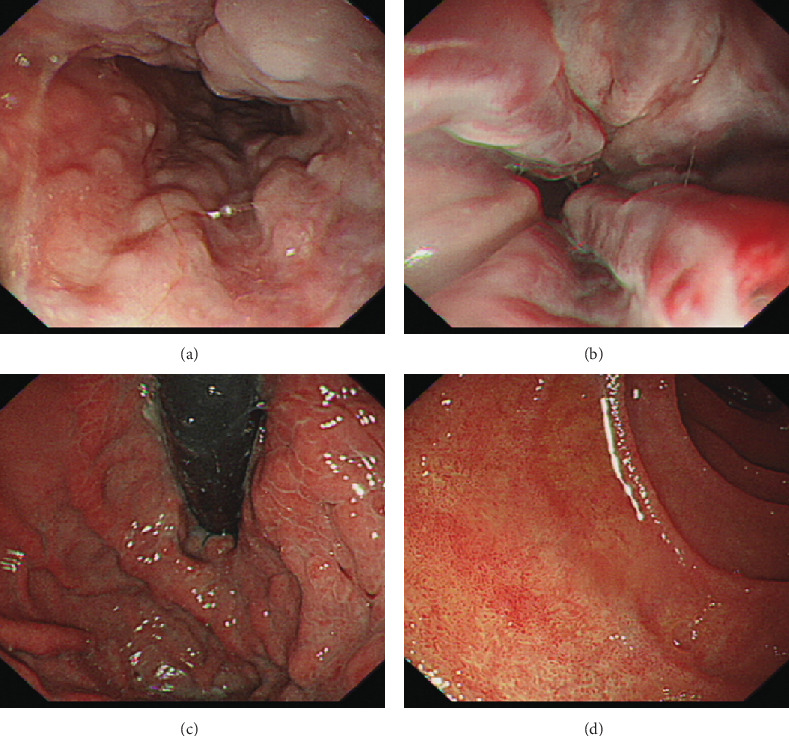
20-month follow-up upper endoscopy showed gradual disappear of gastric varices and F1 esophageal varices.

After hospitalization for GI bleeding, the patient was followed-up in the clinic for the last two years. Dynamics of LFTs and CBC are shown in Table 1. Upper endoscopy during the most recent follow-up in May 2019 showed gradual disappearance of gastric varices and F1 esophageal varices (Figure 4). The patient has no recurrent GI bleeding.

## 4. Discussion

Felty's syndrome (FS) is a potentially serious systemic condition, which is complicated with prolonged rheumatoid arthritis (RA). Neutropenia is the most common and important feature of FS, while splenomegaly is not always present [2]. FS is a very rare complication in RA in Han Chinese patients, with an occurrence of less than 0.1% [9]. HLA-DR4 works as the predisposing genetic background for this disease [10]. Pathophysiology of FS-associated neutropenia includes increased peripheral destruction of neutrophils, failure of bone marrow to produce neutrophils, and neutrophil sequestration in patients with splenomegaly. Recent data showed that anti-G-CSF antibody may contribute to the neutropenia [11]. Methotrexate is widely accepted as the first-line treatment for FS [12]. Hydroxychloroquine was shown to successfully increase the neutrophil count in patients who cannot tolerate methotrexate [13]. Biological agents like rituximab, etanercept, and abatacept showed promising effects in controlling RA symptoms and increasing neutrophil count. However, the cost and side effects of rituximab are major concerns [14–16]. Granulocyte colony stimulating factor (G-CSF) is effective in increasing neutrophil count during severe neutropenia in FS. But G-CSF is associated with flu-like symptoms, vasculitis skin rash, thrombocytopenia, hyperuricemia, and severe bone pain [17–19]. Splenectomy can be avoided in the majority of FS patients since methotrexate and biological agents can effectively manage the severe neutropenia in FS [3, 20]. The cost of the surgery and recurrence of neutropenia postsurgery are major concerns for offering splenectomy to patients.

Portal hypertension is a clinical syndrome that causes the pressure of portal venous system to increase and collateral circulation to open due to obstruction or abnormal increase pressure of portal vein. The most common cause of portal hypertension is liver cirrhosis. Additional causes include idiopathic portal hypertension, pancreatic portal hypertension, portal spongy degeneration, and Budd-Chiari syndrome. About 20% of FS patients develop portal hypertension and esophageal gastric varices during disease progression. Thorne evaluated 18 patients with FS, of which five patients had portal hypertension (including esophageal varices or elevated intrahepatic pressure) [21]. Pathogenesis of portal hypertension in FS patients is controversial. DeCoux et al. and Stock et al. reported cases of FS and suggested that increased spleen blood fluid may contribute to portal hypertension. Short-term follow-up showed improvement of esophageal gastric varices after splenectomy, which further confirmed this opinion [6, 22]. However, about 70% of liver biopsy results of FS patients showed nodular regenerative hyperplasia (NRH). Sweeney suggested NRH could compress intrahepatic venous radicals and sinusoids, leading to portal hypertension [5]. However, NRH is now recognized as a morphological manifestation of PSVD [23]. When we reviewed those reported cases of FS with portal hypertension, we found that majority of the FS patients with portal hypertension fulfill the diagnosis criteria of PSVD [24]. In our patient, the liver biopsy showed disappearance of portal vein in portal area and herniated portal vein, which also indicate diagnosis of PSVD. Besides, the liver stiffness of this patient further supported the diagnosis of PSVD [25]. Taken together, these findings suggested comorbid with PSVD might be one of the causes of portal hypertension in FS patients.

Previous case reports showed that splenectomy reduces portal vein flow and can be effectively used to treat the esophageal gastric varices in FS [6]. However, the number of cases is limited and no long-term follow-up data is available. Also, splenectomy is not generally recommended in management of PSVD [24]. Endoscopic therapy is another option for the prevention of bleeding in these patients [26]. For this patient, we chose endoscopic therapy for the management of varices. Two-year follow-up showed control of varices and no rebleeding.

## 5. Conclusion

In conclusion, we reported an FS patient complicated with esophageal gastric variceal bleeding. Liver pathology indicated PSVD. A series of endoscopic therapy were performed for the control of variceal bleeding successfully, without recurrence of bleeding for two years. The clinical presentation and liver histological features in this patient suggested that the PSVD was a likely cause of portal hypertension. Based on the management experience and outcomes of the present case, endoscopic therapy is recommended for variceal bleeding in FS patients.

## Figures and Tables

**Table 1:** Dynamics of liver function tests, complete blood count, and rheumatoid factors of the patient.

	26/09/2017	12/10/2017	22/01/2018	03/07/2018	03/05/2019
ALT (U/L)	18.8	21	11.9	35.0	77.8
AST (U/L)	17.9	27.4	13.6	32.4	86.1
TBIL (*μ*mol/L)	9.9	9.4	11.8	15.2	23.5
WBC (×10^9^/L)	2.62	2.22	2.55	2.32	2.62
NEU (×10^9^/L)	1.80	1.61	1.83	1.93	2.05
Hb (g/L)	75.0	85.0	109.0	105.0	102.0
PLT (×10^9^/L)	73.0	65.0	60.0	45.3	27.2
RF (IU/mL)	20	NA	NA	NA	1100

NA: not available; RF: rheumatoid factors.

## Data Availability

The data used to support the findings of this study are included within the article.
